# Statin prescription among patients with type 2 diabetes in Botswana: findings and implications

**DOI:** 10.1186/s12902-020-0516-7

**Published:** 2020-03-10

**Authors:** Julius Chacha Mwita, Brian Godman, Tonya M. Esterhuizen

**Affiliations:** 1 Department of Internal Medicine, Faculty of Medicine, University of Botswana, Private Bag, 00713 Gaborone, Botswana; 2 Division of Epidemiology and Biostatistics, Department of Global Health, Faculty of Medicine and Health Sciences, Stellenbosch University, Stellenbosch, South Africa; 3 Department of Laboratory Medicine, Division of Clinical Pharmacology, Karolinska Institutet, Karolinska University Hospital Huddinge, Stockholm, Sweden; 4 Strathclyde Institute of Pharmacy and Biomedical Sciences, University of Strathclyde, Glasgow, G4 0RE United Kingdom; 5 School of Pharmacy, Sefako Makgatho Health Sciences University, Pretoria, South Africa

**Keywords:** Statin, Type 2 diabetes mellitus, Prescription and Botswana

## Abstract

**Background:**

There is evidence of statin benefit among patients with diabetes regardless of cholesterol levels or prior cardiovascular disease history. Despite the evidence, there is under-prescription of statins in clinical practice. This study aimed to assess statin prescriptions and associated factors among patients with type 2 diabetes in Botswana.

**Methods:**

The study was a secondary data analysis of 500 randomly selected type 2 diabetes patients at a specialised diabetes clinic at Gaborone, Botswana. We assessed the proportion of statin-eligible patients who are prescribed statins and evaluated the adjusted associations between various factors and statin prescriptions.

**Results:**

Overall, 477 (95.4%) participants were eligible for a statin prescription. Clinicians prescribed statins in 217 (45.5%) of eligible participants, and only one (4.4%) ineligible participant. The probability of a statin prescription was higher in participants with high baseline low-density lipoprotein cholesterol (risk ratio [RR]: 1.49; 95%CI: 1.17–1.89), increasing duration of diabetes (RR: 1.01; 95%CI 1.00–1.03) and the presence of chronic kidney disease (RR: 1.35; 95%CI: 1.06–1.74).

**Conclusion:**

A large proportion with type 2 diabetes in Gaborone is not receiving statins. Clinicians did not consider most guideline-recommended indications for statin prescriptions. The findings call for improvement in diabetes quality of care by implementing evidence-based guideline recommendations.

## Background

Cardiovascular disease (CVD), which includes coronary artery disease (CAD), cerebrovascular accident (CVA), and peripheral arterial disease (PAD), is common and contributes to over two-thirds of mortality among patients with type 2 diabetes mellitus [1–3]. While the presence of type 2 diabetes alone confers the highest risk for CVD of any single risk factor, the coexistence of other cardiovascular risk factors is a common phenomenon [3, 4]. Consequently, guidelines advise screening and optimal treatment of CVD risk factors in patients with diabetes [5, 6]. Besides, prescribing of 3-hydroxy-3-methylglutaryl-coenzyme A reductase inhibitors (statins) among patients with type 2 diabetes reduces the risk of major CVD events by 23–33% [7–9]. There is evidence of statin benefit among patients with diabetes regardless of their low-density lipoprotein cholesterol (LDL-C) values or prior CVD history [7, 9–14]. For each mmol/l reduction in LDL-C, there is evidence of a 9% relative reduction in all-cause mortality in patients with diabetes [15]. Irrespective of LDL-C, guidelines recommend statins for patients with diabetes aged ≥40 years without atherosclerotic cardiovascular disease (ASCVD), or those who are younger than 40 years but with existing ASCVD or additional risk factors [5, 6].

While some studies in developed countries have reported high use of statins among patients with type 2 diabetes, there has generally been under-prescribing of statins across many countries [16–18]. Statin prescribing among patients with type 2 diabetes is as high as 100% in developed countries but as low as 3–13% in Africa [17–23]. The suboptimal utilisation of statin therapy in Africa is due to many factors, but mainly limited access to standard diabetes care because of the high cost of tests and medications with typically high co-payment levels [22]. Affordability is a critical issue in several African countries where there is no universal healthcare, with the cost of medicines accounting for up to 70% of total healthcare expenditure, much of which is out-of-pocket [24, 25]. Statin under-prescribing is a concern given the high growth rates of cardiovascular diseases in sub-Saharan African countries and current poor control of cardiovascular diseases [26–31]. The underuse of statins significantly increases the incidence of cardiovascular events and associated mortality [32]. Although healthcare is free in Botswana, factors not related to cost may still affect the uptake of statins in patients with diabetes. This is problematic given current prevalence rates of diabetes in Botswana and the resultant impact on morbidity and mortality [33–35]. Published data on the impact of diabetes mellitus on CVD in Botswana are scant. About a quarter of patients admitted with heart failure had type 2 diabetes in a recent study in Gaborone [33]. Although data on atherosclerotic diseases in Botswana are missing, we can expect that diabetes-related coronary atherosclerosis underlines some cases of heart failure [33, 35]. In addition, the rising burden and suboptimal control of CVD risk factors in patients with type 2 diabetes have become an increasing concern in Botswana [36]. One of the critical steps that will prevent or slow ASCVD in this suboptimally-treated patients is the use of statins [5]. Currently, there is no study assessing the extent of statin prescriptions among patients with type 2 diabetes in Botswana. We aimed to address this by evaluating the extent of statin prescription among patients with type 2 diabetes in Botswana. Our secondary aim was to determine factors associated with statin prescription among type 2 diabetes.

## Methods

### Study design

We conducted a secondary analysis of data from a previous study among type 2 diabetics at a specialised diabetes clinic in Gaborone, Botswana. Any concerns with the management of diabetic patients in this dedicated clinic are likely to be exacerbated in non-specialist centres such as primary healthcare centres.

### Participant recruitment and data collection

The original study took place between August 2017 and February 2018 [36]. The primary objective of the original study was to assess glycaemic, low-density lipoprotein, and hypertension control in patients with type 2 diabetes. The study included 500 randomly selected patients with type 2 diabetes aged ≥18 years, attending the clinic for at least 3 months before data collection. Using systematic random sampling, we enrolled every eighth patient from the daily lists of patients attending the clinic. The enrolment occurred in the mornings and afternoons until either the daily target of ten patients was achieved or the end of clinic consultations. Demographic data (age, sex, occupation, educational attainment, and marital status), duration of diabetes, and the type of diabetes medications were collected. Other information was the history of hypertension, lipid disorders, ischemic heart diseases, stroke or peripheral vascular disease. We also recorded data on the use of medications for hypertension and lipid disorders (including statins), and anthropometric measurements (weight, height, hip and waist circumferences). With participants in light clothing and without footwear, we measured participants’ height and weight to the nearest 0.1 cm (cm) and 0.1 kg (kg) respectively [37]. We also measured the hip and waist circumferences (to the nearest 0.1 cm a non-stretchable tape measure at a hip and level midway between the lowest rib and iliac crest [38]. After 10 min of rest, three seated blood pressure (BP) measurements were taken from the right arm using a digital automatic BP monitor (Omron) [37, 39]. We documented the average of the three readings and that of each patient’s previous visit. In addition, we used the electronic medical records to obtain participants’ serum creatinine and low-density lipoprotein-cholesterol (LDL-C), and urine dipsticks for proteinuria results within 6 months of enrolment [36]. The diabetes clinic has a satellite laboratory that performs tests per protocol as defined by the ISO-9000 certified Princess Marina Hospital clinical laboratory. Total cholesterol (TC), triglycerides (TG) and high-density lipoprotein cholesterol (HDL-C) are measured enzymatically in plasma using commercial kits made by Roche Diagnostics, Switzerland. LDL-cholesterol was calculated using the Friedwald formula as TC-HDL-C-TG/2.2 in mmol/dL [40]. For the present study, we evaluated the extent of statin prescriptions among the participants. The primary outcome measure was receiving a statin prescription among statin-eligible participants. We assessed statin eligibility based on the Society for Endocrinology, Metabolism, and Diabetes of South Africa (SEMDSA) guidelines [6]. According to SEMDSA, the eligibility for statin prescribing included any CVD or chronic kidney disease (CKD), age above 40 years, and diabetes duration longer than ten years. In addition, a statin prescription is indicated in the presence of one or more of the following cardiovascular risk factors; hypertension, cigarette smoker, HDL-C level, family history of early CAD, and any albuminuria [6]. Other independent variables included baseline serum LDL-C, body mass index (BMI), waist-hip ratio (WHR) and level of education.

### Definition of terms

The diagnosis of hypertension based on the self-reported history of hypertension, the use of hypertension-lowering medications or sustained blood pressure ≥ 140/90 mmHg in more than one visit [41]. We defined CVD as the history of CAD, CVA (ischemic stroke, transient ischemic attacks), or PAD [6]. CAD was any documented definite or probable myocardial infarction, CAD-related revascularisation (surgery, angioplasty, stenting, or any combination of these), or stable angina in participants’ medical records [42]. Data on CVA and PDA were extracted from participants’ medical records as defined by the treating physician. Smoking status was a documented self-report of current smoking habits. We estimated glomerular filtration rate (eGFR) using the Modification of Diet in Renal Disease(MDRD), and classified patients with eGFR < 60 ml/minute/1.73m^2^ as having CKD [43]. BMI was categorized into underweight for BMI < 18.5 kg/m^2^; normal for BMI of 18.5–24.9 kg/m^2^, overweight for 25.0–29.9 kg/m^2^; or obese for BMI ≥30 kg/m^2^ [44]. We also calculated the WHR by dividing waist circumference (cm) by hip circumference (cm) and defined WHR ≥ 0.85 for women and ≥ 0.90 for men as high [38]. Dipstick proteinuria appeared as negative (−), trace, (+), (++), or (+++) in the dataset. We classified proteinuria in individuals with ≥ (+) dipstick proteinuria results. For patients already on lipid-lowering medications and whose baseline ‘untreated’ levels of lipid profile were not available, we estimated the LDL-C levels before the initiation of statin treatment as in previous studies [45]. The adjustment was made based on the assumption that most patients received atorvastatin (the only statin available in the public sector in Botswana) at a dosage of at least 10 mg per day and an overall adherence of 60% [46]. We calculated the baseline LDL-C levels by assuming that the measured LDL-C is a result of a 25% reduction from baseline [45]. Baseline LDL-C levels above 4.13 mmol/l were considered high [47].

### Statistical analysis

Clean data were imported from MS Excel and analysed using Stata Version 14 (Stata Corp, College Station, TX). Categorical variables were presented as frequencies and percentages, and continuous variables as mean (standard deviation [SD]) or medians [first–third quartiles]. Comparison of clinical and demographic factors by gender and statin use was achieved by using the Chi-square or Fisher’s exact tests for categorical variables, and independent student’s t-tests or Wilcoxon rank-sum test for continuous variables as appropriate. A 2-sided *p*-value < 0.05 was considered as statistically significant. As the outcome of interest (a statin prescription) was a common event, and our study design was cross-sectional, we estimated relative risks (RRs) as measures of association. Log-binomial models or generalised linear models for the binomial family were fitted to assess for independent predictors for statin prescribing. The multivariable model included all factors with *p* < 0.2 on bivariate analysis. We used a backward selection modelling method with probabilities set at 0.05 and 0.1 for inclusion and exclusion; respectively. We report adjusted risk ratios (RRs), 95% confidence intervals (CIs), and *p*-values. With a sample size of 477 statin-eligible participants, we were able to estimate the prevalence of statin use of 13% with a margin of error of 3% on a two-sided alpha level of 0.05 [22].

## Results

There were 500 participants in the dataset, with a mean (SD) age of 58.9 (12.2) years and 330 (66%) were females. Table 1 summarises baseline participants’ characteristics by gender. The majority (96.7%) of participants aged ≥40 years and women were significantly older than men. Approximately a third (34.4%) of participants had a diabetes duration of over ten years. Hypertension (84.7%) and obesity (51.6%) were prevalent, especially in female participants. Overall, CKD (11.3%), proteinuria (10.7%), CVD (8.8%), and smoking (3.4%) were uncommon. The mean (SD) baseline LDL-C was 3.1 (1.2) mmol/L, and significantly higher in female than male participants.

**Table 1:** Demographic and clinical characteristics of patients with type 2 diabetes at a specialised Diabetes clinic in Gaborone (*N* = 477)

**Characteristics**	**All (*N*** **= 477)**	**Males (*n*** **= 160)**	**Females (*n*** **= 317)**
**Age, mean(SD), years**	60.3(10.8)	56.8(11.5)	62.0(10.1)
**Age > 40 years n(%)**	461(96.7)	150(93.8)	311(98.1)
**Diabetes duration, median, IQR, years**	7(3–13)	8.9(3–14)	7 (3–13)
**Diabetes duration > 10 years n(%)**	164 (34.4)	63(39.4)	101(31.9)
**BMI, mean (SD) kg/m**^**2**^	30.7(6.0)	29.0(5.2)	31.6(6.1)
Normal weight n(%)	85(17.8)	39(24.4)	46(14.5)
Overweight n(%)	146(30.6)	59(36.7)	87(27.4)
Obese n(%)	246(51.6)	62(38.8)	184(58.0)
**Marital status**			
Living alone n(%)	247(51.8)	47(29.4)	200(63.1)
Living with a partner n(%)	230(48.2)	113(70.6)	117(36.9)
**Education status**			
≤ Primary education, n(%)	306(64.1)	82(51.3)	224(70.7)
≥ Secondary or tertiary, n(%)	171(35.9)	78(48.7)	93(29.3)
**WHR, mean (SD)**	0.94(0.10)	0.97(0.08)	0.93(0.09)
Low WHR n (%)	79(16.6)	67(41.9)	12(3.8)
High WHR n (%)	398(83.4)	93(58.1)	305(96.2)
**Hypertension n (%)**	404(84.7)	120(75.0)	284(89.6)
**Use of antihypertensive n (%)**	389(81.6)	110(68.8)	279(88.0)
**Smoking n (%)**	16(3.4)	12(7.5)	4(1.3)
**Lipid-lowering medications n (%)**	224(47.0)	70(43.8)	154(48.6)
Statins n (%)	217(45.5)	68(42.5)	149(47.0)
Others n (%)	7(1.5)	2(1.6)	5(1.6)
**CVD n (%)**	42(8.8)	15(9.4)	27(8.5)
PAD n (%)	11(2.3)	3(1.9)	8(2.5)
Coronary artery disease n (%)	12(2.5)	5(3.3)	7(2.2)
Cerebrovascular disease n (%)	22(4.6)	8(5.0)	14(4.4)
**CKD n (%)**	54(11.3)	24(15.0)	30(9.5)
**Proteinuria n (%)**	51(10.7)	27(16.9)	24(7.6)
**HbA1c mean (SD), %**	8.4(2.4)	8.5(2.6)	8.3(2.3)
**Baseline LDL-C, mean (SD), mmol/L**	3.1(1.2)	2.8(1.1)	3.3(1.2)
Normal	315(66.0)	113(70.6)	202(63.7)
High	59(12.4)	9(5.6)	50(15.8)
Missing	103(21.6)	38(23.8)	65(20.5)

Legend: *BMI* Body Mass Index, *CKD* chronic kidney disease, *CVD* cardiovascular disease, *HbA1c* Haemoglobin A1c, *IQR* interquartile range, *LDL-C* low-density lipoprotein cholesterol, *PAD* peripheral artery disease, *SD* standard deviation, *WHR* waist-hip ratio

### Statin eligibility and prescribing rates

Of the 500 participants, 477 (95.4%) were eligible for a statin prescription. Clinicians prescribed statins (exclusively atorvastatin) in 217 (45.5%) of statin-eligible participants, and only one (4.4%) ineligible participant. Seven (1.5%) participants received prescriptions of other lipid-lowering medications alone or in combination with statins. Of those who were eligible for statins, statin-prescribed individuals differed from those without prescriptions in several parameters on the bivariate analysis (Table 2). Relative to the statin-non-prescribed group, the statin-prescribed group had a longer duration of diabetes (8.9 years vs. 6.0 years; *p* < 0.001); were older (61.5 years vs 59.2 years; *p* = 0.018), more likely to be hypertensive (85.7% vs 78.1%; *p* < 0.032), more likely to have CKD (17.2% vs 6.6%; *p* = 0.001), and a higher baseline LDL-C (3.3 vs 2.9 mmol/L; p < 0.001). The two groups did not differ significantly in the presence of CVD, proteinuria and gender.

**Table 2:** Factors associated with statin prescription among statin-eligible patients with type 2 diabetes at a specialised Diabetes clinic in Gaborone (*N* = 477)

**Characteristics**	**Statin not prescribed (*n*** **= 260)**	**Statin prescribed (*n*** **= 217)**	***P*-value**
Sex			
Males, n (%)	92(35.4)	68(31.3)	0.351
Female, n (%)	168(64.6)	149(68.7)	
Diabetes duration, median, IQR, years	6(2–12)	8.9(4–15)	< 0.001
Duration ≤10 years	179(68.9)	134(61.8)	0.104
Diabetes duration > 10 years	81(31.1)	83(38.2)	
Age, mean (SD), years	59.2(10.8)	61.5(10.7)	0.018
Age ≤ 40 years, n (%)	12(4.6)	4(1.8)	< 0.094
Age > 40 years n (%)	248(95.4)	213(98.2)	
Marital status			
Living alone n (%)	135(51.9)	112(51.6)	0.946
Living with a partner n (%)	125(48.1)	105 (48.4)	
Education status			
≤ Primary education, n (%)	166(63.9)	140(64.2)	0.879
≥ Secondary or tertiary, n (%)	94(36.1)	77(35.8)	
Hypertension n (%)	212(81.5)	192 (88.5)	0.036
Antihypertensive use n (%)	203(78.1)	186(85.7)	0.032
Smoking	13(5.0)	3(1.4)	0.029
CVD, n (%)	20(7.7)	22(10.1)	0.538
PAD, n (%)	7(2.7)	4(1.8)	0.625
Coronary artery disease n (%)	6(2.3)	6(2.8)	0.751
Cerebrovascular diseases n (%)	9(3.5)	13(6.0)	0.190
BMI, mean (SD) kg/m^2^	30.3(5.7)	31.2(6.2)	0.113
Normal weight n (%)	50(19.2)	35(16.1)	0.669
Overweight n (%)	79(30.4)	67(30.9)	
Obese n (%)	135(47.9)	115(52.8)	
WHR, mean (SD)	0.94(0.08)	0.95(0.10)	0.106
Low WHR n (%)	46(17.7)	33(15.2)	0.467
High WHR n (%)	214(82.3)	184(84.8)	
CKD, n (%)	20(7.7)	34 (15.7)	0.006
Proteinuria, n (%)	30 (11.5)	21(9.7)	0.512
HbA1c mean (SD), %	8.4(2.6)	8.4(2.2)	0.948
Baseline LDL-C mean (SD), mmol/L	2.9(0.9)	3.3(1.4)	0.003
Normal	177(68.1)	120(55.3)	< 0.001
High	17(6.5)	42(19.4)	
Missing	66(25.4)	55(25.3)	

Legend: *BMI* body mass index, *CKD* chronic kidney disease, *CVD* cardiovascular disease, *HbA1c* haemoglobin A1c, *IQR* interquartile range, *LDL-C* low-density lipoprotein cholesterol, *PAD* peripheral artery disease, *SD* standard deviation, *WHR* waist-hip ratio

### Multivariable analysis

The multivariable log-binomial model examined adjusted associations between statin prescription and various factors. The best fit had the following covariates: age, the duration of diabetes, BMI, hypertension, a high baseline LDL-C, CKD, CVD, and proteinuria. Increasing diabetes duration was associated with an increased likelihood (RR: 1.01; 95%CI 1.00–1.03) of receiving a statin prescription (Table 3). The presence of CKD (RR: 1.35; 95%CI: 1.06–1.74) and a high baseline LDL-C (RR: 1.49; 95%CI: 1.17–1.89) were also associated with an increased likelihood of a statin prescription. Age, BMI, history of CVD, and a diagnosis of hypertension were not associated with statin prescribing after adjustment for the other variables in the model.

**Table 3:** Adjusted relative risks for associations between various factors and statin prescription among statin eligible patients with diabetes at a specialised diabetes clinic in Botswana

**Characteristic**	**Risk ratio**	**95% Conf. Interval**	***p*-value**
Age, years	1.006	0.994–1.017	0.362
CKD	1.354	1.055–1.738	0.017
Hypertension	1.336	0.846–2.110	0.213
BMI, kg/m^2^	1.014	0.994–1.034	0.16
High baseline LDL	1.488	1.173–1.887	0.001
Diabetes duration, years	1.014	1.000–1.027	0.048
Proteinuria	0.979	0.644–1.488	0.922
CVD	0.901	0.623–1.303	0.581

Legend: *BMI* body mass index, *CKD* chronic kidney disease, *CVD* cardiovascular disease, *LDL-C* low-density lipoprotein cholesterol

## Discussion

Less than half of the statin-eligible patients with type 2 diabetes at a specialised diabetes clinic in Botswana received a statin prescription. A longer duration of diabetes, a higher baseline LDL-C and the presence of chronic kidney disease were independently associated with the tendency to prescribe statins.

The under-prescription of statins in this population is a concern since the use of statins appreciably reduces cardiovascular morbidity and mortality in patients with diabetes irrespective of their LDL-C levels [7, 9–13]. Whilst the proportion of patients with diabetes who are prescribed statins varies substantially worldwide; there is a low prescribing of statins both in developing and developed countries [18, 19, 21–23, 48–51]. Encouragingly, the percentage of patients with diabetes who received statins (45.5%) in our study appears appreciably higher than the 3–13% seen in some African countries and consistent with findings from developed countries where between 25 to 73% of patients with diabetes are prescribed statins [18, 19, 22, 48–50]. The proportion of statin prescription in this population is higher than has been reported in some developed countries, including Germany (25%) and the United Kingdom (33%) [18, 48]. While the finding of a comparatively higher statin prescription in this setting than some African countries and some developed countries is encouraging, there is no reason for complacency as more than half of our patients were without CVD protection by statins. Similar to developed countries, one potential explanation for low statin prescribing rates among our patients with diabetes is inadequate adherence to guidelines [22, 51–53]. Whereas there may be a fear of the association of statin therapy with a slightly increased risk of developing diabetes, the benefits of statins in reducing cardiovascular morbidity and mortality among patients with established diabetes should dispel these concerns [7–9, 54]. As mentioned, several epidemiological studies have observed a lower proportion of statin prescribing in patients with diabetes in Africa than in this population [21–23]. In addition to inadequate adherence to guidelines, the main reasons for low statin prescribing in Africa include limited access to these medicines due to their high cost with high co-payment levels, inaccessibility to lipid testing facilities and unavailability of guidelines [22]. The presence of free consultations, tests and medications in Botswana might explain our higher statin prescribing rates than those seen in other African settings without universal health access. Irrespective of the reasons, it is imperative that statins are routinely prescribed to reduce the risk of CVD events in patients with type 2 diabetes [7–13, 15, 55].

Our results of increasing statin prescribing with increasing diabetes duration also agree with previous research findings [56]. This finding is reassuring as a longer duration of diabetes leads to an increased risk of CVD. For this reason, guidelines recommend statins for patients with diabetes for more than ten years [5, 6]. Although the median diabetes duration was seven years in our participants, the association between statin prescribing and diabetes duration was still apparent. The finding may suggest that clinicians correctly recognise a longer duration of diabetes as an indication for statin therapy. As it may take time for the transmission of information between clinicians and patients, the acceptance of new medications is likely easier as the diabetes duration increases [57].

Another finding in our study was that the presence of CKD increased the likelihood of statin prescribing. This finding is also encouraging as statins reduce mortality by up to 36% in patients with kidney failure [5, 6, 58, 59]. The finding is also consistent with SEMDSA guideline recommendations of a statin for every patient with diabetes and CKD [6]. While albuminuria is a marker of renal disease, participants with proteinuria did not receive statin prescriptions. We can postulate that clinicians do not recognise proteinuria as a predictor of CVD and an indication for statins in patients with diabetes. This finding warrants further investigation as it is in contrast with Berthold et al., who reported increased odds of statin prescribing in type 2 diabetes patients with proteinuria in Germany [18].

Our findings that a high baseline LDL-C increased the likelihood of statin prescribing agreed with those of Berthold at al. that showed an 11% increase in statin prescribing rates for every 0.26 mmol/L increase in LDL-C [18]. This finding also confirms the observation from previous studies that prescribers tend to respond more to the pre-treatment LDL-C value than to the patients’ overall CVD risk profile as described in clinical guidelines [9, 52]. Although there is a lack of local guidelines, our diabetes clinic adopted the SEMDSA guidelines which recommend statins along with lifestyle changes regardless of cholesterol levels for all patients with diabetes aged > 40 with or without CVD [6]. Our findings that statin prescription was based on LDL-C level may suggest a need for deliberate efforts for improving the understanding and implementation of the adopted guidelines, and we will be taking this further.

In most clinical guidelines, the presence of CVD, CKD, patients age, diabetes and presence of CVD risk factors such as hypertension, albuminuria and cigarette smoking are indicators of prescribing statins among patients with type 2 diabetes [5, 6]. The presence of any of the above factors is associated with an increased risk of CVD. Except for CKD and duration of diabetes, none of the other indications was a predictor of statin prescriptions in this population. Given the high prevalence of hypertension and other indications in this population, most participants would have qualified for statins if guideline recommendations were followed. As our clinic has adopted the SEMDSA guidelines, this finding is a concern and a call for efforts to improve its implementation for the benefit of this high-risk population.

We are aware of a number of limitations of our study. We estimated the baseline LDL-C levels by a 25% adjustment of measured LDL. There was a risk of either overestimation or underestimation of the baseline LDL-C due to possible errors in our assumptions of the dosage and adherence of atorvastatin. Although measured LDL-C results were available for most of the included participants, HDL cholesterol results were mostly missing. Guidelines consider HDL as one of the factors for statin prescribing in patients with diabetes. However, data for all other indications for statin prescribing were available in dataset. We did not document the dosage of statin used in our patients; hence, we are unable to determine whether moderate to high-intensity statins were prescribed as recommended by the guidelines. In addition, we were unable to identify patients with contraindications to warfarin as the information was not available. The study was also performed in one clinic, hence limiting the generalizability of the study findings to other facilities in the country. However, being one of the few specialised diabetes clinics in Botswana, our results likely characterise the ‘finest’ diabetes care in the country. Consequently, the highlighted concerns are likely to be greater in non-specialist healthcare facilities treating patients with type 2 diabetes in Botswana.

## Conclusion

In conclusion, we believe this study provides a useful and reliable picture of current statin prescribing behaviour in Botswana despite the limitations. There is appreciable under-prescribing of statins in this high-risk population. The presence of CKD, high baseline LDL, and an increased duration of diabetes strongly influenced statin prescriptions in patients with diabetes. Clinicians did not consider most guideline-recommended indications for the prescribing of statins. By identifying gaps in the prescription of statins to patients with diabetes, the study provides an opportunity for improvement in the quality of care. Furthermore, the study findings suggest a need for further studies to investigate the reasons for statin under-prescription in our setting. We are following this up to provide future guidance for clinicians in Botswana treating patients with type 2 diabetes, with the results likely to be of interest to other sub-Saharan African countries with high rates of type 2 diabetes.

## Data Availability

The datasets used and/or analysed during the current study are available from the corresponding author upon reasonable request and with permission of the HRDC of Botswana Ministry of Health and Wellness.

## Abbreviations

ASCVD: atherosclerotic cardiovascular disease
BMI: Body mass index
CAD: coronary artery disease
CKD: chronic kidney disease
CVD: Cardiovascular disease
eGFR: estimated glomerular filtration rate
HDL-C: HbA1c: Haemoglobin A1c
HDL-C: High-density lipoprotein cholesterol
HRDC: Health Research Development Committee
LDL-C: Low-density lipoprotein cholesterol
MDRD: Modification of Diet in Renal Disease
PAD: peripheral artery disease
SEMDSA: Society for Endocrinology, Metabolism, and Diabetes of South Africa
WHR: Waist-Hip ratio
